# Omics community detection using multi-resolution clustering

**DOI:** 10.1093/bioinformatics/btab317

**Published:** 2021-05-11

**Authors:** Ali Rahnavard, Suvo Chatterjee, Bahar Sayoldin, Keith A Crandall, Fasil Tekola-Ayele, Himel Mallick

**Affiliations:** Department of Biostatistics and Bioinformatics, Computational Biology Institute, Milken Institute School of Public Health, The George Washington University, Washington, DC 20052, USA; Epidemiology Branch, Division of Intramural Population Health Research, Eunice Kennedy Shriver National Institute of Child Health and Human Development, National Institutes of Health, Bethesda, MD 20892, USA; School of Systems Biology, College of Science, George Mason University, Fairfax, VA 22030, USA; Department of Biostatistics and Bioinformatics, Computational Biology Institute, Milken Institute School of Public Health, The George Washington University, Washington, DC 20052, USA; Epidemiology Branch, Division of Intramural Population Health Research, Eunice Kennedy Shriver National Institute of Child Health and Human Development, National Institutes of Health, Bethesda, MD 20892, USA; Biostatistics and Research Decision Sciences, Merck & Co., Inc, Rahway, NJ 07065, USA

## Abstract

**Motivation:**

The discovery of biologically interpretable and clinically actionable communities in heterogeneous omics data is a necessary first step toward deriving mechanistic insights into complex biological phenomena. Here, we present a novel clustering approach, *omeClust*, for community detection in omics profiles by simultaneously incorporating similarities among measurements and the overall complex structure of the data.

**Results:**

We show that *omeClust* outperforms published methods in inferring the true community structure as measured by both sensitivity and misclassification rate on simulated datasets. We further validated *omeClust* in diverse, multiple omics datasets, revealing new communities and functionally related groups in microbial strains, cell line gene expression patterns and fetal genomic variation. We also derived enrichment scores attributable to putatively meaningful biological factors in these datasets that can serve as hypothesis generators facilitating new sets of testable hypotheses.

**Availability and implementation:**

*omeClust* is open-source software, and the implementation is available online at http://github.com/omicsEye/omeClust.

**Supplementary information:**

Supplementary data are available at *Bioinformatics* online.

## 1 Introduction

Finding biologically meaningful groups exhibiting coherent within-group similarities and between-group differences is often the first critical step in any analysis of modern high-throughput data. An interesting characteristic that real biological networks represent is the clustering or community structure, under which the network topology is organized into modules commonly known as communities or clusters. While clustering and community discovery differ in their representation of detected group entities, they share many commonalities and our use case in this study is based on biological questions benefiting both finding clusters and detecting communities.

Despite being a highly researched unsupervised problem supported by a myriad of algorithms from diverse scientific disciplines, clustering and community structure detection remains computationally and biologically challenging. This is particularly due to the technical nature of the associated data, which are typically noisy and high dimensional with confounding effects unique to individual technology (e.g. platform-specific batch effects). With ever-increasing multi-omics efforts and the associated technology-specific challenges, there is a need for more data-driven methods that are capable of finding biologically meaningful communities in a technology-agnostic manner.

In addition to the omics-specific challenges, there are some long standing issues shared by most existing clustering (Altman, 1992; Sibson, 1973) and community detection algorithms (Blondel *et al.*, 2008; Bohlin *et al.*, 2014). Clear challenges remain to determine the most appropriate number of clusters as well as the most appropriate distance metric for a particular dataset. For example, a problem akin to hierarchical clustering is the careful tuning of the resolution parameter as well as the selection of appropriate evaluation criteria. When applied in high-throughput biology contexts, these are exacerbated by sparse and highly variable measurements with additional challenges introduced by the heterogeneity of the associated features. For instance, in metabolomics, some groups of metabolites have greater inter-feature distances than others or in microbial communities, distance between species is often related to sample environment (Lloyd-Price *et al.*, 2017). These, together with the inter-individual differences introduced by the potential confounding factors (e.g. gender and age), calls for an algorithm that detects communities and attributes putatively meaningful biological factors to the detected community structure.

## 2 Materials and methods

**Fig. 1.**
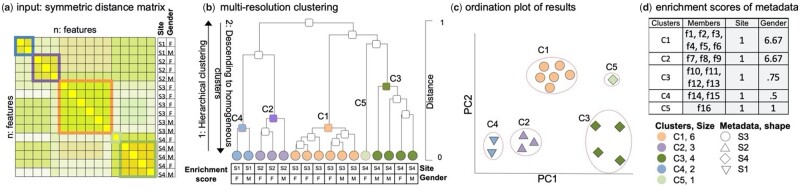
omeClust overview workflow. (**a)** A distance matrix is used as input. The distance matrix can be obtained using any appropriate metric, such as Bray–Curtis distance for microbiome data or nucleotide-based distance for DNA sequence data. (**b**) Two-stage cluster detection applies (i) hierarchical clustering to find the hierarchical distance structure among features and (ii) trace up-down using a binary-silhouette score to find cluster nodes. (**c**) omeClust provides several ordination plots to represent the clustering results. The color represents cluster membership; the shape is used to present the most influential metadata in the detected clusters. (**d)** A tab-delimited file with clusters as rows and feature members, resolution scores for all metadata sorted by the mean of resolution scores for all clusters, and enrichment scores for each metadata are returned in columns

Multi-resolution clustering (*omeClust*) identifies communities within datasets potentially consisting of heterogeneous ‘features’. Features can be biomarkers from omics molecular profiles (e.g. taxa, genes, pathways, chemicals, etc.) potentially accompanied by the associated metadata (e.g. epidemiological variables, clinical, pharmaceutical and environmental covariates, among others). The relevant features are therefore, specifically defined in a study-specific manner relative to the data and research question posed by the individual study. To achieve generality of our approach, a key *omeClust* input is a distance matrix of features. For an input dataset containing values from samples alongside a pre-calculated distance matrix between points (measurements) (Fig. 1a), the *omeClust* algorithm proceeds by (i) building a representation of the overall structure of point distances (a hierarchy) using hierarchical clustering (zoom out) (Fig. 1b), (ii) descending the hierarchy to find heterogeneous clusters (zoom in) using a **binary-silhouette score**, (iii) calculating resolution scores (defined as the harmonic mean of the number of cluster members and the similarity between cluster members) for each cluster to prioritize important clusters and **enrichment scores** including normalized mutual information (NMI) and frequency-based scores for each metadata to rank the influence of the variable on the detected communities (if provided) and (iv) finally, generating graphic visualizations (Fig. 1c and Supplementary Fig. S1) and reporting interpretable clustering results (Fig. 1d). We cover each of these key workflow elements in more detail below.


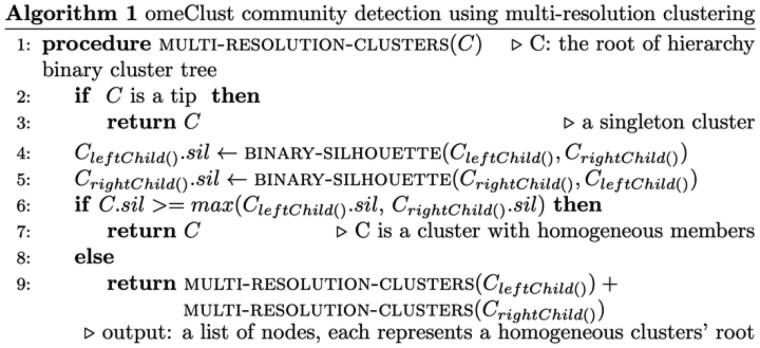


In summary, *omeClust* finds communities by generating an overall structure of relationships between points (samples or features) in the form of a hierarchical cluster based on a user-defined distance matrix, and then descends the hierarchy to find communities of homogeneous features. This approach uses two unique algorithms: (i) Algorithm 1 is designed to descend a hierarchy and return intermediate nodes in the hierarchy that represent communities, and (ii) Algorithm 2 is similarly designed to find heterogeneous clusters using a binary-silhouette score.

### 2.1 Binary-silhouette score

Binary-silhouette, defined in Algorithm 2, is a measurement to quantify how well members within a branch are related compared to the sibling branch in a hierarchy. A hierarchy is a binary tree, and each node has two children nodes. We calculate a binary-silhouette score, which is similar to a silhouette score (Ogbuabor and Ugwoke, 2018) except that, in binary-silhouette scoring, we only consider two clusters, left and right, rather than all clusters, when the score is measured. When we measure the binary-silhouette score for the left child (*from*: a) in a node, we use the immediate right child as another cluster (*to*: b) and vice-versa.


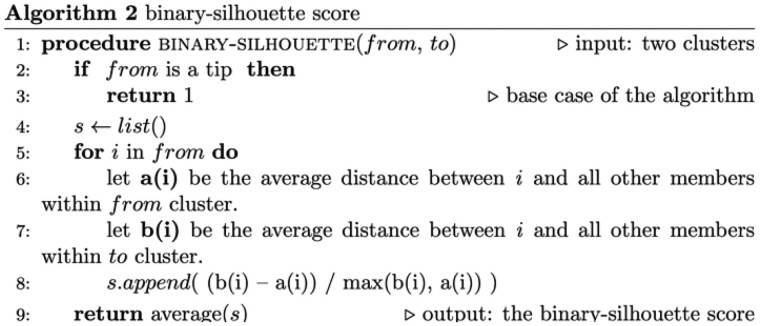


### 2.2 Enrichment score

To measure the influence of each metadatum on the community structure, *omeClust* implements two approaches, ***freq*** and ***nmi***. While *omeClust* by default, discretizes the continuous metadata, essentially any metadata that can be converted to a float data type can be used as an input to the *omeClust* algorithm. For the ***freq***approach, enrichment scores are computed by calculating the number of occurrences of the most frequent category of a metadata in a community, which is then scaled by dividing by the number of community members. The mean enrichment scores for each major cluster are then used to rank the influence of each metadatum on the detected community structure. Major clusters with >0.05 enrichment scores are highlighted and returned. The ***nmi*** approach, on the other hand, directly uses the NMI (Estévez *et al.*, 2009; Kvålseth, 2017) between the community labels and the metadata values as enrichment scores. NMI is a measure to evaluate the dependency between two variables, and it is a variant of mutual information from information theory which provides an interpretable quantification. If two variables are completely independent (no association) the NMI will be 0, whereas, for two identical variables, the NMI will be 1.

At a deeper level, *omeClust* uses hierarchical clustering to build an overall structure of potentially heterogeneous features and subsequently detects communities of related features. For hierarchical clustering, by default, *omeClust* uses a complete-linkage approach (Großwendt and Röglin, 2017); however, the algorithm is robust to the specification of the linkage method (Supplementary Fig. S2) as other well-used approaches, such as *single*, *average*, *complete*, *weighted*, *centroid*, *median* and *ward* can be provided as options by the user. Specifically, for community detection, *omeClust* undertakes a top-down recursive approach beginning at the root of the hierarchical tree and descending to a set of nodes within the tree. For descent, *omeClust* compares a node’s binary-silhouette score with its two direct children. This procedure is repeated until termination, i.e. when the selected nodes represent single features in their respective data trees or the current nodes’ binary-silhouette score is larger than its children. All tips under a node in the hierarchy are considered a cluster if the node has a greater score than its children. Philosophically, *omeClust’*s approach is similar to a static tree cut (e.g. *the cutree* function in R) that creates groups from hierarchical clustering using an arbitrary cut threshold and returns groups in branches under the cut level (Langfelder *et al.*, 2008). However, *omeClust’*s approach differs fundamentally from *cutree* in that *omeClust* finds the cut levels adaptively at various distance levels, whereas *cutree* cuts all the hierarchy at an arbitrary and constant level provided by the user.

## 3 *OmeClust* increases community detection power in omics data

When applied to datasets with no clear cluster structure, *omeClust* reports singleton clusters, as expected, ruling out the possibility of data artifacts and false positive findings (results not shown). To determine *omeClust*’s ability to recapitulate true positive clusters, we further validated *omeClust* on datasets with known cluster structure (detailed in Section 3.1). Since *omeClust* coherently looks at multiple resolution levels of the underlying hierarchical structure, it is expected to discover clusters (communities) above and beyond those detectable only by computing average distances between clusters. This is particularly relevant for omics data, where large groups of molecular features (e.g. expression values for genes in a metabolic pathway) form communities with variable mean distances between members and it is desirable to incorporate the entire resolution spectrum of the intra-cluster distances into the clustering algorithm.

### 3.1 Synthetic data validation of *omeClust*

To evaluate *omeClust*, we tested our algorithm along with existing state-of-the-art approaches (You and You, 2018) in a variety of synthetic datasets containing built-in known communities (‘ground truth’) and evaluated their statistical and computational performance across a range of parameters. For generating synthetic clusters with ‘ground truth’ membership information, we used *clusterlab* (John *et al.*, 2020), which is a recent method that allows simulation of Gaussian clusters (Maugis *et al.*, 2009) with controlled spacing, size and variance among the generated clusters. Specifically, we generated 135 synthetic datasets of varying capacity across a range of pairwise combinations of cluster size (4, 6, 8) and per-cluster sample size (10, 20, 40), while also varying the feature dimensions (500, 1000, 1500) and inter-cluster distances (0.05, 0.10, 0.25, 0.5, 1).

**Fig. 2.**
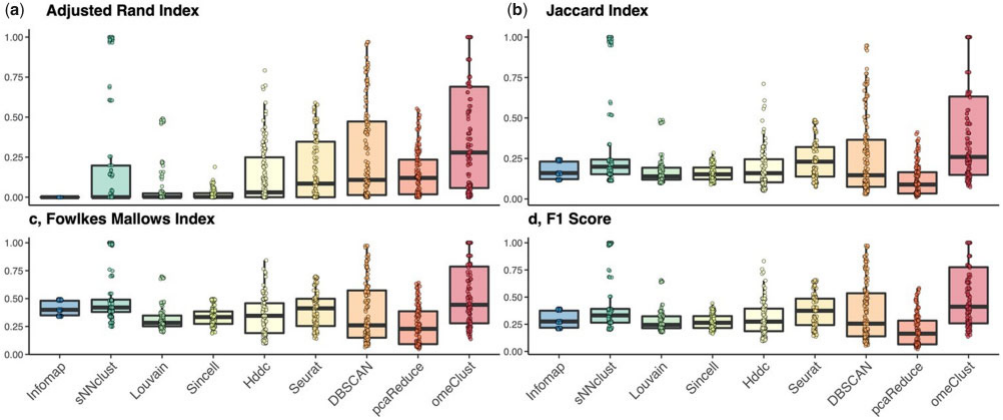
*omeClust* improves community detection power. 135 synthetic datasets were generated using *clusterlab (*John *et al.*, 2020) with varying number of clusters (*k*=4, 6, 8), cluster sizes (*n*=10, 20, 30), feature dimensions (*P*=500, 1000 and 1500) and distances among clusters (alpha =0.05, 0.1, 0.25, 0.5, 1) measured against a variety of indices with scores from 0 to 1, with 0 indicating identical features and 1 indicating features with random similarity. (**a**) *omeClust* has a higher adjusted Rand index as compared to nine other clustering methods displaying greater degree of agreement among partitions and/or clusters given the ground truth information. (**b**) *omeClust* similarly has a higher Jaccard index as compared to existing methods displaying greater degree of similarity given the ground truth information. (**c)** In terms of Fowlkes–Mallows index, *omeClust* is also one of the best-performing methods displaying greater degree of similarity between its clustering set and the ground truth information. (**d)** Finally, *omeClust* has a higher *F*1 score as compared to all other clustering methods displaying a better weighted average between precision and recall given the ground truth information

We found that *omeClust* vastly outperforms popular community detection methods, such as the Louvain approach (Blondel *et al.*, 2008) and Infomap (Bohlin *et al.*, 2014) in terms of (i) the adjusted Rand index (Fig. 2a), (ii) Jaccard index (Fig. 2b), (iii) Fowlkes–Mallows index (Fig. 2c) and (iv) *F*1 score (Fig. 2d). While these network community detection approaches tend to perform well in readily distinguishable communities, they suffer in the presence of inherent multicollinearity, noise and overlap as typically observed in omics samples and can in turn give rise to misleading communities (Supplementary Fig. S1). In addition to superseding these more sophisticated methods and improving community detection, *omeClust* further exhibited improved performance as compared to several other state-of-the-art domain-agnostic clustering algorithms, such as the partitional [e.g. Sincell (Juliá *et al.*, 2015), pcaReduce (Žurauskienė and Yau, 2016) and Seurat (Satija *et al.*, 2015)], network-based [e.g. Infomap (Bohlin *et al.*, 2014) and Louvain (Blondel *et al.*, 2008; Csardi *et al.*, 2006)], model-based [e.g. Hcmodel (Fraley *et al.*, 2014)], density-based [e.g. DBSCAN (Liu *et al.*, 2007)], subspace-based [e.g. Hddc (Bouveyron *et al.*, 2007; Bergé *et al*., 2013)] and shared nearest neighbor-based [e.g. sNNclust (Ertöz *et al.*, 2003) and sscClust (Ren *et al.*, 2019)] community detection methods [Supplementary Tables S1 and S2 and Fig. 2(a–d)]. The superior performance of *omeClust* remained consistent across a range of feature dimensions, inter-cluster distances and linkage methods, which further highlights the flexibility and robustness of *omeClust* in realistically unbalanced community structures. Taken together, these findings confirm that by taking into account the overall high-level structure of diverse features, in addition to feature-wise distances, which alone may not be sufficient to reproducibly recover biologically complex communities, *omeClust* is able to capture biologically relevant communities, missed by other methods, across a broad range of realistically complex scenarios.

### 3.2 Empirical validation and application of *omeClust*

#### 3.2.1 Niche association of human microbial species and strains

**Fig. 3.**
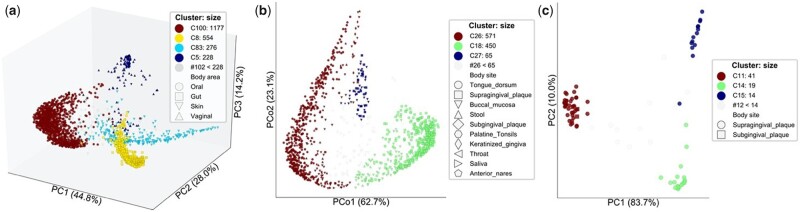
*omeClust reveals biologically meaningful clusters of omics features*. (**a**) *omeClust* detects four major communities based on Bray–Curtis dissimilarity in human-associated microbial species, finding strong segregation by body area (i.e. oral, gut, skin and vaginal) revealing site-specific subspecies clades and dynamics across major body site habitats. (**b)** *H.parainfluenzae* strain clades are detected as communities of microbial strains using a combination of HMP1-II and iHMP data using Kimura two-parameter distance (Kimura, 1980). This reveals that microbial species not only have different strain subclades in different niches, but also can have more than one strain in each sample. (**c**) *omeClust* detected three communities of *A.johnsonii* mostly detected in supragingival plaque body site

We first applied *omeClust* to 2484 metagenomes from the expanded human microbiome project (HMP1-II) (Lloyd-Price *et al.*, 2017). In this application, the ‘features’ included the microbial speices inferred from the metagenomic data (Franzosa *et al.*, 2018) as well as clinical metadata for each sample (including body area of the collection site). *omeClust* identified body area as the most influential metadata (NMI=0.83) responsible for the clustering structure (Fig. 3a). *omeClust* also reports four major clusters (resolution score >0.05) each corresponding to a human body site from which samples were collected, confirming the compositional structure of microbial species and their niche associations. Two metadata, RANDSID (random ID for patients) and SNPRNT (specimen barcode ID), had the lowest frequency-based enrichment scores (<0.05), as expected. The overlaps of color and shape explain how well our computational approach defines the underlying clustering structure, providing actionable interpretations of the detected clusters or communities. Several ordination plots, such as principal coordinates analysis, *t*-distributed stochastic neighbor embedding and multidimensional scaling were used to visualize the results. We also combined HMP1-II samples with the iHMP (Lloyd-Price *et al.*, 2019) shotgun metagenomic data on the human stool samples, and profiled additional 1149 microbial strains that passed the strain conditions (Truong *et al.*, 2017). *omeClust* detected three communities of *Haemophilus parainfluenzae* with body site being the most influential metadata, also confirmed with an independent PERMANOVA analysis (Anderson, 2017) using the *adonis* R function in the *vegan* package (*P*-value = 0.001 with 999 permutations). These communities largely correspond to two body sites, supragingival plaque (two communities) and tongue dorsum (one community) with NMI of 0.49 (Fig. 3b). Our novel findings thus suggest that microbial species can have more than one strain in each sample. We detected similar patterns for oral microbial species, including *Actinomyces johnsonii* (Fig. 3c), *Rothia mucilaginosa*, *Campylobacter showae* and *Porphyromonas sp oral taxon 279*, and stool microbial species including *Eubacterium siraeum* and *Escherichia coli* that showed evidence of multiple subclades of strains consistent with the literature (Supplementary Fig. S3).

#### 3.2.2 Cross-tissue analysis of gene and protein expression in cell lines

**Fig. 4.**
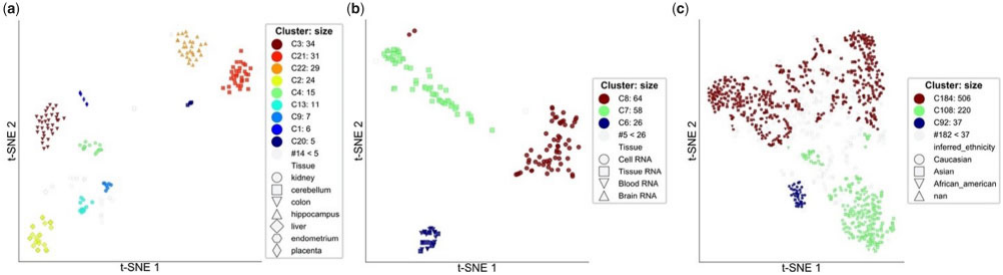
*omeClust with low resolution on gene expression reveals communities of genes that are related to sample tissues. omeClust* provides different ordination plots as exploratory figures to help users visualize the underlying structure of omics data. (**a**) Number of detected communities matches with the number of cell lines with the exception of kidney and cerebellum, where two communities are detected, suggesting possible differences in gene expression between the left and right kidney and cerebellum. However, two communities from the same organs are more similar to each other compared to other organs. (**b**) Proteins are used to find communities of samples, and here we detected three communities with overlap in tissues. (**c**) Using host transcriptome (196 520 gene expression for 1019 samples) we found three communities that inference ethnicity was the most influential metadata; however, the structure in the data was not explained by any major metadata element

Cell line gene expression has been extensively used to investigate intracellular activities in diseases, such as cancer (Ghandi *et al.*, 2019) and inflammatory bowel disease (Schulze *et al.*, 2008). We applied *omeClust* to gene expression datasets from three breast cancer studies (McCall *et al.*, 2011, 2014; Zilliox and Irizarry, 2007). In this application, the omics ‘features’ are gene expression and the clinical metadata include cell line type (i.e. kidney versus cerebellum) and spatial information (whether the sample is collected from the left or right side). Cell line gene expression serves as a strong application to validate our technique as we hypothesized these samples to be clustered according to the kind of tissue from which they originated. *omeClust*, indeed, found that cells establish communities based on their drawn tissues. Using the high-resolution mode of *omeClust*, we detected nine communities from seven tissue cell lines with NMI of 0.84 (Fig. 4a). Cell lines from the kidney and cerebellum formed two communities suggesting spatial heterogeneity of these organs (e.g. whether samples have been collected from the left or right side). Although we did not have spatial resolution data to validate this information, this leads to an interesting hypothesis of spatial gene expression patterns of the left and right side of the kidney and cerebellum, which has been indicated in previous animal studies (Evans *et al.*, 2018; Nakamura *et al.*, 2006). This finding also highlights the versatility of *omeClust*'s clustering capabilities, suggesting that depending on the biological signal present in the dataset, the user may choose the appropriate resolution level. The ability to use multiple resolutions within *omeClust* allows users the flexibility to interpret communities at multiple vantage points (e.g. specific communities associated with different sets of metadata at various resolution levels).

We also used a dataset of 154 samples with 2845 proteins from the Human Protein Atlas database (Uhlén *et al.*, 2015) and found that samples for Cell RNA, Blood RNA and Brain RNA fall in separate communities, although, tissue RNA falls in two clusters: Blood RNA and Brain RNA (Fig. 4b), suggesting that tissue RNA has a broader transcriptomic community and could potentially have similar communities as Blood RNA or Brain RNA. In addition, our results using gene expression from 196 520 genes for 1019 cell lines from the CCLE (Cancer Cell Line Encyclopedia) (Ghandi *et al.*, 2019) show that not one major metadatum explains the structure underlying the data. In particular, inferred ethnicity (Fig. 4c), histology, pathology and gender were the most influential metadata (in decreasing order) and the least enriched metadata in the communities corresponded to the ID variables (i.e. name and depMapID), as expected. This testing strategy is very similar in spirit to a standalone approach like PERMANOVA, where it is possible to attribute the percentage of variation in the distance matrix explained by a metadata of interest. However, unlike PERMANOVA, *omeClust* explicitly uses the clustering and community results to derive the enrichment scores, providing a sophisticated and biologically balanced information for further follow-up experimentation.

#### 3.2.3 Genetic relatedness in fetal growth outcomes

**Fig. 5.**
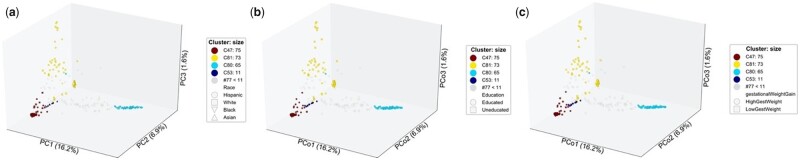
*omeClust reveals communities in fetal growth study.* (**a**) Genome-wide autosomal SNP data were measured for 301 samples from pregnant women in the NICHD Fetal Growth Studies (Grewal *et al.*, 2018). The snpgdsGRM function implemented in SNPRelate (Zheng *et al.*, 2012) was used to calculate the genetic relationship matrix using the correlation method for SNP genotype data between the samples. *omeClust* detected four communities and, Maternal Race is the most influential metadata (NMI=0.51) indicating differences in size of babies at birth for black mothers as compared to other races. (**b**) Maternal socio-economic status (education) was the second most influential metadata (NMI=0.16) indicating differences in the socio-economic status for mothers in the black community influencing the size of babies at birth as compared to other races. (**c**) Maternal cardiometabolic factors (gestational weight gain) was the third most influential metadata indicating differences in cardiometabolic activities of black mothers relating to disproportionate size of babies at birth as compared to other races (NMI=0.12)

Finally, we applied *omeClust* to the NICHD Fetal Growth Studies-Singletons (Grewal *et al.*, 2018) data that included placental samples at delivery from 301 pregnant women from four race/ethnic groups including non-Hispanic white (25.6%), non-Hispanic black (23.9%), Hispanic (33.9%) and Asian/Pacific Islander (16.6%). The samples were genotyped to obtain fetal single nucleotide polymorphisms (SNPs) using HumanOmni2.5 Beadchips (Illumina Inc, San Diego, CA) with ∼2 million SNPs (Delahaye *et al*., 2018). In this application, the SNP data are the genomic ‘features’ and the associated clinical metadata include birth weight, prenatal environment, race, education, etc. Using genetic relationship information among the SNPs as a measure of distance, we set out to detect communities with higher affinity to babies with extremely small or large weight at birth. To this end, we considered several prenatal environments (e.g. maternal age, socio-economic status and parity, among others) as well as low birth weight status (defined as birth weight <2500 g), small for gestational age defined as birth weight less than the 10th percentile for gestational age based on sex-specific birth weight references, and large-for-gestational age defined as birth weight greater than the 90th percentile for gestational age based on sex-specific birth weight references as metadata in the *omeClust* analysis. *omeClust* reported four major clusters primarily overlapping with self-identified race/ethnicity (Fig. 5a). The top three most influential metadata were maternal demographic factor (race), socio-economic status (education) and cardiometabolic factors (gestational weight gain), consistent with previous evidence for population differences in size at birth (Buck Louis *et al.*, 2015; Tekola-Ayele *et al.*, 2018; Tekola‐Ayele *et al*., 2019) [Fig. 5(b–c)]. Specifically, the ordination plots revealed that babies born to self-identified Black mothers had disproportionately smaller weight at birth compared to other races. These findings demonstrate the ability of *omeClust* to cluster samples based on genetic relatedness information and integrate clinical metadata yielding results consistent with findings from published epidemiological studies.

## 4 Discussion

*omeClust* represents a newly developed method to detect clusters and communities in heterogeneous biological datasets. Its validation and applications show that *omeClust* is well suited for finding biologically meaningful subsets of samples or features in a diverse range of omics studies. Key to our approach is the use of the overall structure of the feature-wise relationships in a dataset that allows capturing biologically relevant communities and clusters in different resolutions of similarity. We optimized and validated *omeClust* using realistic synthetic datasets of known community structure, where our approach outperformed existing approaches across a range of scenarios. Notably, *omeClust* remains one of the best-performing methods in the scRNASeq-specific evaluation despite not being optimized for the specific application domain.*omeClust* can also be used for specific downstream tasks, such as discretizing omics data, dimension reduction, microbial beta-diversity analysis and subclade finding in microbial strains using nucleotide-based distances, among others. Further, our approach can be paired with existing network analysis and community detection methods. For example, *omeClust* can find an optimal threshold for sparse edge discovery that can be embedded in a network analysis approach, such as *Infomap* and *Louvain* to improve their performance. Further, *omeClust* outputs an enrichment score for each desired metadata that can be used to select the most influential features in any clustering analysis. This arguably leads to more interpretable communities that are potentially explainable by a few metadata, which can also be used as covariates in streamlined downstream discovery (e.g. differential expression and abundance analysis).

Clearly, the development of robust computational and statistical methods for accurate community detection is an ongoing effort. We, therefore, hope that future work could further fine-tune the task of feature selection and dimensionality reduction based on detected communities (e.g. feature engineering and feature extraction for deep learning). Another opportunity for future extension of our method is allowing multiple omics simultaneously especially allowing multiple time points and tissues to comprehensively detect communities in tandem. Combined, such extensions will allow researchers to use other downstream methods in parallel with *omeClust*, moving toward a robust, unified framework for omics-driven biomarker discovery, development and validation. We believe that the *omeClust* framework and the improved detection of communities represent an important step in this direction that can ultimately aid in better interpretation and understanding of omics data while also encouraging further methodological advances in this area. An open-source (Python) implementation of *omeClust* is freely available at http://github.com/omicsEye/omeClust along with documentation, demo datasets, real-world applications and a user forum.

## Author contributions

A.R. conceived the method; A.R. implemented, packaged and provided online documents and software; S.C., A.R. and H.M. prepared synthetic data and evaluated the performance; B.S., A.R., S.C., and F.T.-A. applied the method on real-world applications. A.R., H.M. and S.C. wrote the manuscript. All authors discussed the results and commented on the article.

## Funding

This work was supported by the National Science Foundation [grant DEB-2028280 to A.R. and K.A.C]. The NICHD Fetal Growth Studies was funded by the Intramural Research Program of the *Eunice Kennedy Shriver* National Institute of Child Health and Human Development, National Institutes of Health including American Recovery and Reinvestment Act funding via contract numbers HHSN275200800013C, HHSN275200800002I, HHSN27500006, HHSN275200800003IC, HHSN275200800014C, HHSN275200800012C, HHSN275200800028C, HHSN275201000009C and HHSN27500008.

*Conflict of Interest*: H.M. is employed by Merck Sharp & Dohme Corp., a subsidiary of Merck & Co., Inc., Kenilworth, NJ, USA. The remaining authors declare that the research was conducted in the absence of any commercial or financial relationships that could be construed as a potential conflict of interest.

## Supplementary Material

btab317_Supplementary_Data

## References

[btab317-B1] Altman N.S. (1992) An introduction to kernel and nearest-neighbor nonparametric regression. Am. Stat., 46, 175–185.

[btab317-B2] Anderson M.J. (2017) Permutational multivariate analysis of variance (PERMANOVA). Wiley StatsRef: Statistics Reference Online. pp. 1–15.

[btab317-B3] Bergé L. et al (2013) Hdclassif: high dimensional supervised classification and clustering. R Package Version, 1, 2.

[btab317-B4] Blondel V.D. et al (2008) Fast unfolding of communities in large networks. J. Stat. Mech. Theory Exp., 2008, P10008.

[btab317-B5] Bohlin L. et al (2014) Community detection and visualization of networks with the map equation framework. In: Ding Y. et al (eds) Measuring Scholarly Impact: Methods and Practice. Springer International Publishing, Cham, pp. 3–34.

[btab317-B6] Bouveyron C. et al (2007) High-dimensional data clustering. Comput. Stat. Data Anal., 52, 502–519.

[btab317-B7] Buck Louis G.M. et al (2015) Racial/ethnic standards for fetal growth: the NICHD fetal growth studies. Am. J. Obstet. Gynecol., 213, 449.e1–449.e41. 10.1016/j.ajog.2015.08.032 PMC4584427 26410205

[btab317-B8] Csardi G. et al (2006) The igraph software package for complex network research. InterJ. Complex Syst., 1695, 1–9.

[btab317-B9] Delahaye F. et al (2018) Genetic variants influence on the placenta regulatory landscape. PLoS Genet., 14, e1007785. 30452450 10.1371/journal.pgen.1007785 PMC6277118

[btab317-B10] Ertöz L. et al (2003) Finding clusters of different sizes, shapes, and densities in noisy, high dimensional data. In: Proceedings of the 2003 SIAM International Conference on Data Mining. Society for Industrial and Applied Mathematics, Philadelphia, PA.

[btab317-B11] Estévez P.A. et al (2009) Normalized mutual information feature selection. IEEE Trans. Neural Netw., 20, 189–201. 19150792 10.1109/TNN.2008.2005601

[btab317-B12] Evans L.C. et al (2018) Transcriptomic analysis reveals inflammatory and metabolic pathways that are regulated by renal perfusion pressure in the outer medulla of Dahl-S rats. Physiol. Genomics, 50, 440–447. 29602296 10.1152/physiolgenomics.00034.2018 PMC6032288

[btab317-B13] Fraley C. et al (2014) mclust: normal mixture modeling for model-based clustering, classification, and density estimation. R Package Version, 4.

[btab317-B14] Franzosa E.A. et al (2018) Species-level functional profiling of metagenomes and metatranscriptomes. Nat. Methods, 15, 962–968. 30377376 10.1038/s41592-018-0176-y PMC6235447

[btab317-B15] Ghandi M. et al (2019) Next-generation characterization of the Cancer Cell Line Encyclopedia. Nature, 569, 503–508. 31068700 10.1038/s41586-019-1186-3 PMC6697103

[btab317-B16] Grewal J. et al (2018) Cohort profile: NICHD fetal growth studies-singletons and twins. Int. J. Epidemiol., 47, 25. 29025016 10.1093/ije/dyx161 PMC5837516

[btab317-B17] Großwendt A. , Röglin H. (2017) Improved analysis of complete-linkage clustering. Algorithmica, 78, 1131–1150.

[btab317-B18] John C.R. et al (2020) M3C: monte Carlo reference-based consensus clustering. Sci. Rep., 10, 1–14. 32020004 10.1038/s41598-020-58766-1 PMC7000518

[btab317-B19] Juliá M. et al (2015) Sincell: an R/Bioconductor package for statistical assessment of cell-state hierarchies from single-cell RNA-seq. Bioinformatics, 31, 3380–3382. 26099264 10.1093/bioinformatics/btv368 PMC4595899

[btab317-B20] Kimura M. (1980) A simple method for estimating evolutionary rates of base substitutions through comparative studies of nucleotide sequences. J. Mol. Evol., 16, 111–120. 7463489 10.1007/BF01731581

[btab317-B21] Kvålseth T.O. (2017) On normalized mutual information: measure derivations and properties. Entropy, 19, 631.

[btab317-B22] Langfelder P. et al (2008) Defining clusters from a hierarchical cluster tree: the Dynamic Tree Cut package for R. Bioinformatics, 24, 719–720. 18024473 10.1093/bioinformatics/btm563

[btab317-B23] Liu P. et al (2007) VDBSCAN: varied density based spatial clustering of applications with noise. In: 2007 International Conference on Service Systems and Service Management. Chengdu, China, pp. 1–4.

[btab317-B24] Lloyd-Price J. et al (2017) Strains, functions and dynamics in the expanded Human Microbiome Project. Nature, 551, 256. 10.1038/nature24485 PMC7608344 29022944

[btab317-B25] Lloyd-Price J. et al (2019) Multi-omics of the gut microbial ecosystem in inflammatory bowel diseases. Nature, 569, 655–662. 31142855 10.1038/s41586-019-1237-9 PMC6650278

[btab317-B26] Maugis C. et al (2009) Variable selection for clustering with Gaussian mixture models. Biometrics, 65, 701–709. 19210744 10.1111/j.1541-0420.2008.01160.x

[btab317-B27] McCall M.N. et al (2011) The Gene Expression Barcode: leveraging public data repositories to begin cataloging the human and murine transcriptomes. Nucleic Acids Res., 39, D1011–D1015. 21177656 10.1093/nar/gkq1259 PMC3013751

[btab317-B28] McCall M.N. et al (2014) The Gene Expression Barcode 3.0: improved data processing and mining tools. Nucleic Acids Res., 42, D938–D943. 24271388 10.1093/nar/gkt1204 PMC3965035

[btab317-B29] Nakamura J. et al (2006) Stomach-selective gene transfer following the administration of naked plasmid DNA onto the gastric serosal surface in mice. Biol. Pharm. Bull., 29, 2082–2086. 17015955 10.1248/bpb.29.2082

[btab317-B30] Ogbuabor G. , Ugwoke F.N. (2018) Clustering algorithm for a healthcare dataset using silhouette score value. Int. J. Comput. Sci. Inf. Technol., 10, 27–37.

[btab317-B31] Ren X. et al (2019) SSCC: a novel computational framework for rapid and accurate clustering large-scale single cell RNA-seq data. Genomics Proteomics Bioinformatics, 17, 201–210. 31202000 10.1016/j.gpb.2018.10.003 PMC6624216

[btab317-B32] Satija R. et al (2015) Spatial reconstruction of single-cell gene expression data. Nat. Biotechnol., 33, 495–502. 25867923 10.1038/nbt.3192 PMC4430369

[btab317-B33] Schulze H.A. et al (2008) From model cell line to in vivo gene expression: disease-related intestinal gene expression in IBD. Genes Immun., 9, 240–248. 18340362 10.1038/gene.2008.11

[btab317-B34] Sibson R. (1973) SLINK: an optimally efficient algorithm for the single-link cluster method. Comput. J., 16, 30–34.

[btab317-B35] Tekola-Ayele F. et al (2018) High burden of birthweight-lowering genetic variants in Africans and Asians. BMC Med., 16, 70. 29792231 10.1186/s12916-018-1061-3 PMC5967042

[btab317-B36] Tekola-Ayele F. et al (2019) Sex differences in the associations of placental epigenetic aging with fetal growth. Aging, 11, 5412–5432. 31395791 10.18632/aging.102124 PMC6710059

[btab317-B37] Truong D.T. et al (2017) Microbial strain-level population structure and genetic diversity from metagenomes. Genome Res., 27, 626–638. 28167665 10.1101/gr.216242.116 PMC5378180

[btab317-B38] Uhlén M. et al (2015) Proteomics. Tissue-based map of the human proteome. Science, 347, 1260419. 25613900 10.1126/science.1260419

[btab317-B39] You K. , You M.K. (2018) Package ‘mclustcomp’.

[btab317-B40] Zheng X. et al (2012) A high-performance computing toolset for relatedness and principal component analysis of SNP data. Bioinformatics, 28, 3326–3328. 23060615 10.1093/bioinformatics/bts606 PMC3519454

[btab317-B41] Zilliox M.J. , Irizarry R.A. (2007) A gene expression bar code for microarray data. Nat. Methods, 4, 911–913. 17906632 10.1038/nmeth1102 PMC3154617

[btab317-B42] Žurauskienė J. , Yau C. (2016) pcaReduce: hierarchical clustering of single cell transcriptional profiles. BMC Bioinformatics, 17, 140. 27005807 10.1186/s12859-016-0984-y PMC4802652

