# Supplementary material for: Omics community detection using multi-resolution clustering
Source: Bioinformatics. 2021 May 11;37(20):3588–94. doi: 10.1093/bioinformatics/btab317 (PMC8545346; doi:10.1093/bioinformatics/btab317)
Supplement: btab317_Supplementary_Data [file btab317_supplementary_data.docx]

**Supplementary materials**

**Omics community detection using multi-resolution clustering**

Ali Rahnavard^1,*^, Suvo Chatterjee^2^, Bahar Sayoldin^3^, Keith A. Crandall^1^, Fasil Tekola-Ayele^2^, Himel Mallick^4,*^

^1^Computational Biology Institute, Department of Biostatistics and Bioinformatics, Milken Institute School of Public Health, The George Washington University, Washington, DC 20052

^2^Epidemiology Branch, Division of Intramural Population Health Research, Eunice Kennedy Shriver National Institute of Child Health and Human Development, National Institutes of Health, Bethesda, MD 20892, USA

^3^School of Systems Biology, George Mason University, Fairfax, VA 22030, USA

^4^Biostatistics and Research Decision Sciences, Merck & Co., Inc., Rahway, NJ 07065, USA

*Correspondence to [rahnavard@gwu.edu](mailto:rahnavard@gwu.edu) and himel.mallick@merck.com

**Implementation and availability.** The details of the *omeClust* algorithm are explained in the following sections including data input format and output explanation. Software documentation, demonstration data, read-application data, and implementation details are available at<https://github.com/omicsEye/omeClust>.

**Data for microbial species and strains.** We used the species-level taxonomy abundances and clinical phenotype information of 2,355 metagenomic samples from the expanded NIH Human Microbiome Project (HMP1-II)[(Lloyd-Price *et al.*, 2017)](https://paperpile.com/c/610DeM/4HCit) to investigate the role of the most influential metadata on the microbial community structure. Microbiome samples were collected from diverse body sites from 242 individuals, with up to three time points per person. Whole metagenomics shotgun sequencing reads from these samples were taxonomically and functionally profiled by HUMAnN2[(Franzosa *et al.*, 2018)](https://paperpile.com/c/610DeM/WUQs). We further applied StrainPhlAn[(Truong *et al.*, 2017)](https://paperpile.com/c/610DeM/2NsM) to get a dominant strain representative in each sample, in which we used the multi sequencing alignment (MSA) files of samples for strains representatives of each species and used Kimura two-parameter distance[(Kimura, 1980)](https://paperpile.com/c/610DeM/uzaL) to measure dissimilarity between strains. To focus our analysis on the specific species of interest, we used HMP1-II oral microbial strains, and additionally, for *Haemophilus parainfluenzae,* which is both oral and gut species, we combined the HMP1-II and iHMP[(Lloyd-Price *et al.*, 2019)](https://paperpile.com/c/610DeM/lqJS) metagenomic samples to investigate the subspecies specialization of this species across body sites.

**Fetal growth study cohort.** The current study included women who participated in the NICHD Fetal Growth Studies-Singletons, a longitudinal study of fetal growth among 2802 pregnant women without major pre-existing medical conditions[(Grewal *et al.*, 2018)](https://paperpile.com/c/610DeM/QLpe). The NICHD Fetal Growth Studies recruited women without major pre-existing medical conditions from 12 clinic sites in the United States that represented four self-identified race/ethnic groups (i.e., non-Hispanic White, non-Hispanic Black, Hispanic, and Asian or Pacific Islander) and followed them through delivery. The present analysis included 301 women who provided placental samples and passed quality control. The 301 women self-identified their race/ethnicity as non-Hispanic white (25.6%), non-Hispanic black (23.9%), Hispanic (33.9%), and Asian/Pacific Islander (16.6%). Women were on average (mean±SD) 27.7±5.3 years old and delivered at 39.5±1.1 gestational weeks. Size at birth was recorded and 77% of the women gave birth to appropriate gestational age (AGA) babies while 19% gave birth to small for gestational age (SGA) and 4% gave birth to large for gestational age (LGA) babies[(Tekola-Ayele *et al.*, 2019)](https://paperpile.com/c/610DeM/xRpa). The study was approved by institutional review boards at NICHD and each of the participating clinical sites. Written informed consent was obtained from each woman who participated in the study.

**Placental genotyping in fetal growth data.** Placental DNA samples were genotyped using HumanOmni2.5 Beadchips (Illumina Inc., San Diego, CA), followed by initial data processing using Illumina’s GenomeStudio, as previously described[(Delahaye *et al.*, 2018)](https://paperpile.com/c/610DeM/xtVc). SNPs were excluded if they had excessive missing genotype (SNPs with genotype call rate of < 95%), deviated from Hardy-Weinberg equilibrium (p value < 0.0001), and had low minor allele frequency (< 0.05). A total of 11 samples showing discrepancies between phenotypic sex and genotypic sex (n = 4), that were outliers from the distribution of the samples genetic clusters based on multi-dimensional scaling plots (n = 6), and with a mismatching sample identifier (n = 1) were excluded.

**Cell line gene expression data.** The CCLE (Cancer Cell Line Encyclopedia) data includes genetic and pharmacologic characterization for over 1100 cell lines. 196520 gene expressions have been recorded in the current dataset. The order of influence of metadata were: inferred_ethnicity, Histology, Pathology, Gender, Site_Primary, Race, Original.Source.of.Cell.Line, Hist_Subtype1, type_refined, type, tcga_code, PATHOLOGIST_ANNOTATION, Freezing.Medium, Site_Of_Finding, Characteristics, Growth.Medium, Disease, Age, Supplements, Site_Subtype1, Doubling.Time.Calculated.hrs Doubling.Time.from.Vendor, mutRate, Name, depMapID.

**Synthetic data generation and evaluation.** A recently developed method *clusterlab*[*(John et al., 2020)*](https://paperpile.com/c/610DeM/cBik) was used to simulate multivariate Gaussian clusters to represent data bearing similarities to high-dimensional omics datasets. Briefly, the algorithm first creates N points equally spaced on the circumference of a circle in 2D space and these form the centers of each cluster to be simulated. Additional samples are added by adding Gaussian noise to each cluster center and concatenating the new sample coordinates. Together, these aspects make *clusterlab* a flexible Gaussian cluster simulation tool with precise control over the size, variance, and spacing of the clusters in NXN dimensional space. Taking advantage of such key features in *clusterlab*, we simulated 135 synthetic datasets with varying cluster size (4, 6, 8) and per-cluster sample size (10, 20, 40), while also varying the feature dimensions (500, 1000, 1500) and inter-cluster distances (0.05, 0.10, 0.25, 0.5, 1). To evaluate the performance of *omeClust*, several external validation metrics were considered given the ground truth knowledge such as i) Adjusted Rand Index, ii) Jaccard Index, iii) Fowlkes-Mallows Index and iv) F1 score. We implemented these evaluation metrics using the *mclustcomp()* function in the *mclustcomp*[*(You and You, 2018)*](https://paperpile.com/c/610DeM/9Ecf) R package . To rank cluster, resolution score is defined as harmonic mean of number of cluster members and similarity among theme using 1.0 - the cluster’s condensed distance in the hierarchy.


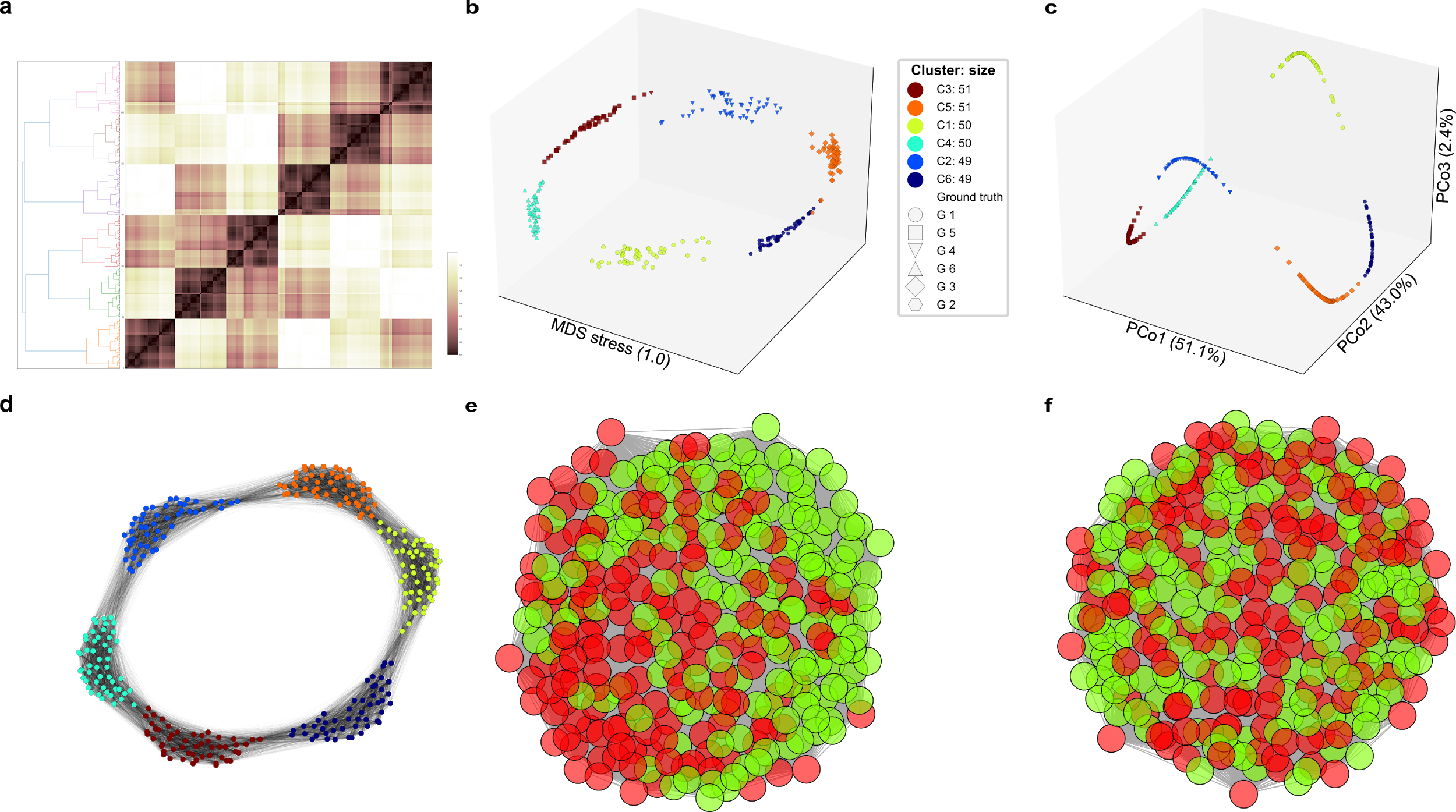


**Supplementary Figure 1: *omeClust* produces various plots to visualize the results for ease of interpretation. a-d**, dendrogram with heatmap, multidimensional scaling (MDS), Principal coordinate analysis (PCoA), and network visualization are examples of *omeClust* results visualization on synthetic data of 300 samples and 1000 features with 6 clusters. *omeClust* results match with ground truth given as metadata to be visualized. Color represents clusters found by *omeClust*, and shape reflects the ground truth of data given as the metadata input to *omeClust*. **e**. the results of the Louvain approach, and **f**, shows results from Infomap. Infomap and Louvain find two communities displayed in red and green colors.


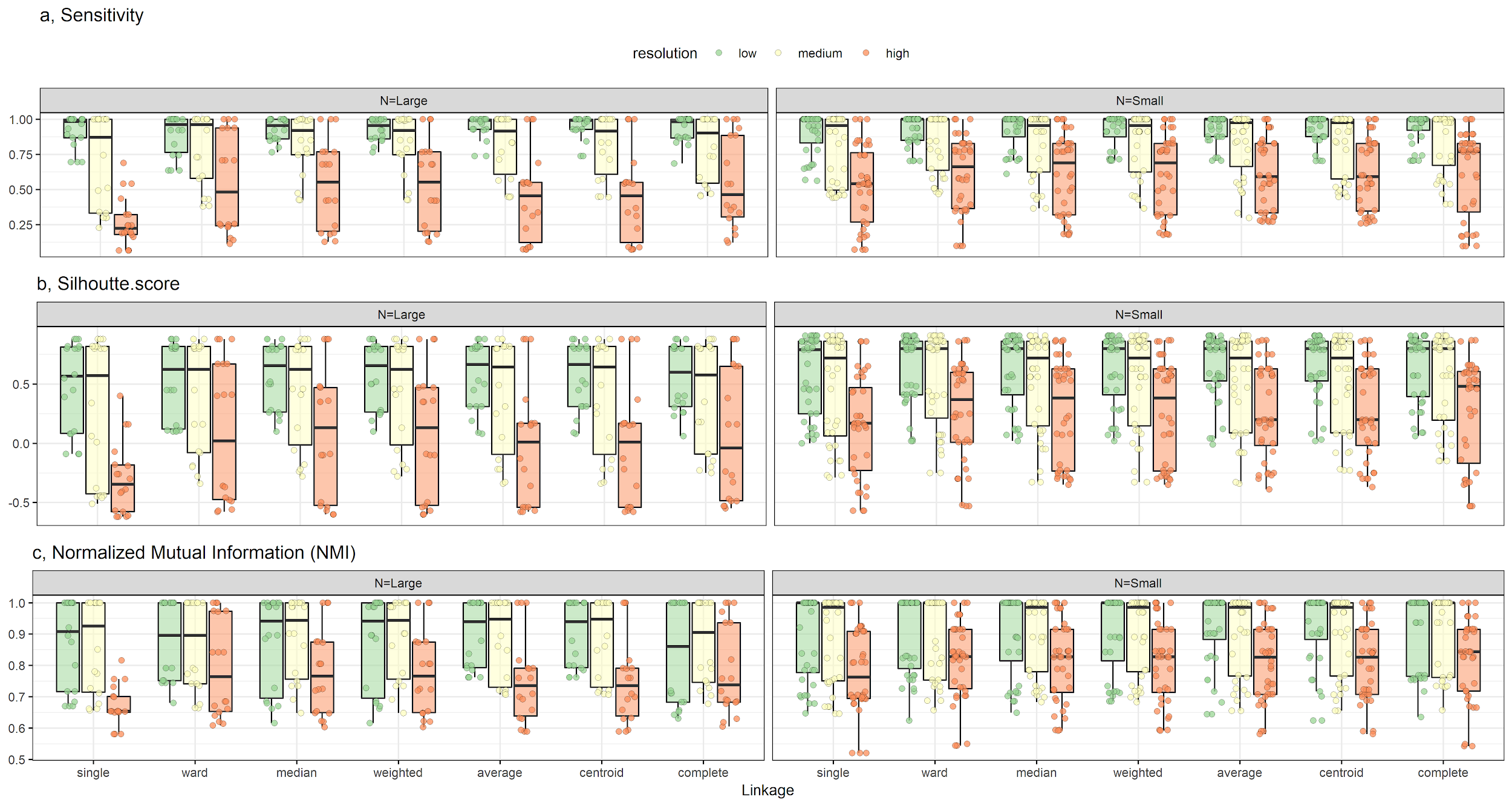


**Supplementary Figure 2: Benchmarking reveals that *omeClust* is robust to linkage and sample size variations.** To evaluate the best configuration of *omeClust* under the influence of different sample sizes, linkage methods, and resolutions, we implemented *omeClust* to 2 sample size categories, small (n= 40, 60, 80) and large (n = 120, 180, 240) along with 7 different linkage methods and 3 different resolutions (low, medium, and high) available with *omeClust*. Among all combinations, low resolution and complete linkage consistently outperformed the other combinations. **a**, Sensitivity in all scenarios has a higher value in low resolution, and high resolution gives us detailed smaller clusters but sensitivity is lower. **b**, Silhouette score drops in high resolution due to breakdown of cluster to subcluster. **c**, normalized mutual information (NMI) as a measure to find similarity between detected communities and ground truth communities has a higher value in low resolutions across all linkage methods. Overall *omeClust* performs similarly for linkage methods which further highlights the overall behavior of *omeClust* using a combination of two steps: building hierarchical clusters and then descend to find homogeneous clusters. Overall, complete linkage shows consistently better performance.


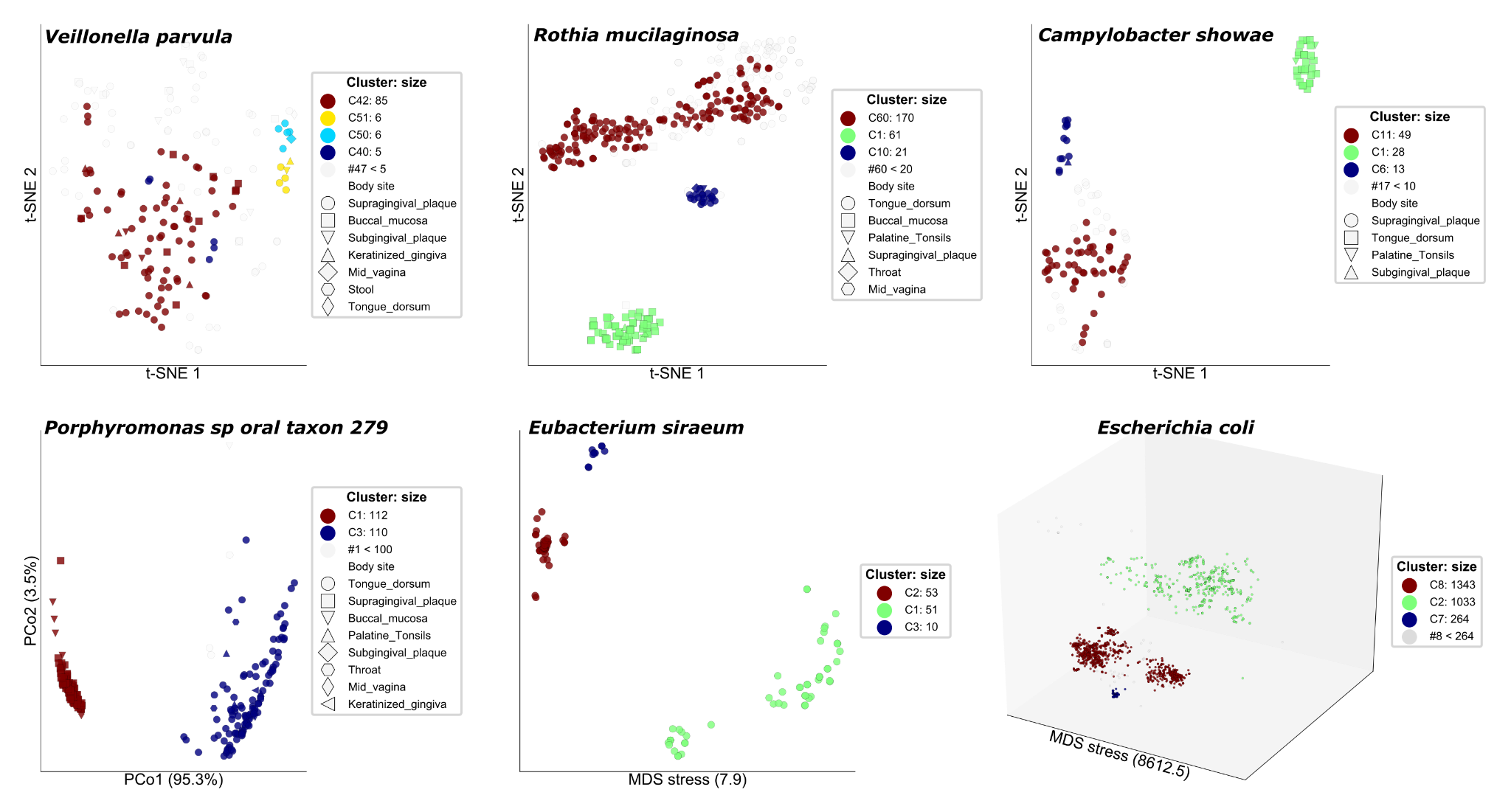
**Supplementary Figure 3:** ***omeClust* quantifies niche-association of human oral microbial strains.** Distances among human microbiome strains using the Kimura 2-parameter distances is used to investigate strain subclades of microbial species associated with metadata (eg. human body sites). *Veillonella parvula* does not show community structure across body sites, however, microbial species such as *Rothia mucilaginosa*, *Campylobacter showae*, *Porphyromonas sp oral taxon 279*, *Eubacterium siraeum*, *Escherichia coli*. This reveals strains with strain-specific subclades, possessing different dynamics between the microbial strains’ subclades. each have three strains subclades associated with oral body sites, Tongue dorsum (two communities in this body site) and Buccal mucosa. omeClust detected 3 communities of *Rothia mucilaginosa,* andCampylobacter showae, corresponding to two body sites Supragingival plaque (two communities) and Tongue dorsum (one community). *Porphyromonas sp oral taxon 279* has two communities corresponding to strains subclades in two bodysites, Tongue dorsum and Buccal mucosa.

| **Method Type** | **Algorithm** | **Library (function) [platform]** |
| --- | --- | --- |
| Model-based: Gaussian Mixtures | Hcmodel | mclust (hc) [R][(Fraley *et al.*, 2014)](https://paperpile.com/c/610DeM/umtK) |
| Density-based | DBSCAN | dbscan (dbscan) [R][(Liu *et al.*, 2007)](https://paperpile.com/c/610DeM/FvaZ) |
| Subspace-based | Hddc | HDclassif (hddc) [R][(Bergé *et al.*, 2013)](https://paperpile.com/c/610DeM/BKQC) |
| Graph-based: Community Detection | Louvain | igraph(louvain) [R][(Csardi *et al.*, 2006)](https://paperpile.com/c/610DeM/H8fO) |
| Graph-based: Community Detection | Infomap | igraph(louvain) [R][(Csardi *et al.*, 2006)](https://paperpile.com/c/610DeM/H8fO) |
| Graph-based: Shared nearest neighbor | SNNclust | dbscan (sNNclust) [R][(Ertöz *et al.*, 2003)](https://paperpile.com/c/610DeM/fOA0) |

**Supplementary Table 1:** Summary of domain-agnostic clustering techniques. Method names along with their corresponding algorithmic details and computing platforms are provided.

| **Method** | **Type** | **Algorithm** | **Platform** |
| --- | --- | --- | --- |
| Seurat[(Satija *et al.*, 2015)](https://paperpile.com/c/610DeM/ux1H) | graph-based | PCA + KNN + Louivan | R |
| Sincel[(Juliá *et al.*, 2015)](https://paperpile.com/c/610DeM/1mjQ)l | Partition | K-mediods | R |
| pcaReduce[(Žurauskienė and Yau, 2016)](https://paperpile.com/c/610DeM/fiQj) | Linkage/Partition | PCA + k-means + hierarchical | R |
| sscClust[(Ren *et al.*, 2019)](https://paperpile.com/c/610DeM/Zee4) | graph-based | Shared nearest neighbor | R |

**Supplementary Table 2:** A summary of domain-specific clustering techniques for single-cell RNA sequencing data. Method names along with their corresponding algorithmic details and computing platforms are provided.
